# Long-term outcomes of patients with single ventricle who do not undergo Fontan palliation^☆^

**DOI:** 10.1016/j.ijcchd.2023.100457

**Published:** 2023-04-05

**Authors:** Wayne C. Zheng, Yves d’Udekem, Leeanne E. Grigg, Dominica Zentner, Rachael Cordina, David S. Celermajer, Edward Buratto, Igor E. Konstantinov, Melissa G.Y. Lee

**Affiliations:** a Heart Research, Clinical Sciences, Murdoch Children's Research Institute, Melbourne, Australia; b Department of Cardiac Surgery, The Royal Children's Hospital, Melbourne, Australia; c Department of Paediatrics, University of Melbourne, Melbourne, Australia; d Department of Cardiology, Alfred Health, Melbourne, Australia; e Division of Cardiac Surgery, Children's National Hospital, Washington, United States; f Department of Cardiology, The Royal Melbourne Hospital, Melbourne, Australia; g Department of Medicine, The Royal Melbourne Hospital, University of Melbourne, Melbourne, Australia; h Department of Cardiology, Royal Prince Alfred Hospital, Sydney, Australia

**Keywords:** Arrythmia, Fontan, Heart failure, Mortality, Outcomes, Single ventricle

## Abstract

**Background:**

Patients with single ventricle (SV) without Fontan palliation are uncommon, and their long-term outcomes remain unclear.

**Methods:**

Retrospective study of 35 adult patients with SV without Fontan from two tertiary centers. Primary outcome was mortality.

**Results:**

Median age at first follow-up was 31 years (IQR: 20–40). Most common defect was double inlet left ventricle (34%), and 69% had left ventricular morphology. Patients were unoperated (46%), had systemic-to-pulmonary artery shunt (31%) or bidirectional cavopulmonary shunt (23%) as final palliation. Most common reasons for not progressing to Fontan palliation were pulmonary vascular disease (54%), patient refusal (17%), Fontan takedown (14%), and hypoplastic pulmonary arteries (11%). Baseline mean hemoglobin was 195 ± 29 g/L, mean O_2_ saturation 83 ± 6.9%, and 4 patients in NYHA Class III‒IV. After a mean follow-up of 10 ± 8.3 years, there were 9 deaths with heart failure being the leading cause (n = 6). Age-adjusted survival of these adult SV survivors was 73% and 53% at 40 and 50 years of age, respectively. Deceased patients more frequently had renal impairment (50% vs 0%) and QRS prolongation (75% vs 16%) at baseline (all p < 0.05). During follow-up, 40% had a new arrhythmia (atrial: n = 14, ventricular: n = 3), 34% had one or more hospitalizations for heart failure, and 17% had a stroke. A greater proportion of patients with pre-existing or new atrial/ventricular arrhythmia died compared to those without (42% vs 6%, p = 0.02).

**Conclusions:**

Patients with SV without Fontan have high mortality and a substantial burden of cardiovascular complications, particularly arrhythmia. QRS prolongation and renal impairment were associated with mortality.

## Abbreviations

ACHD: Adult Congenital Heart Disease
BCPS: Bidirectional cavopulmonary shunt
BMI: Body mass index
CHD: Congenital heart disease
CMR: Cardiac magnetic resonance imaging
DILV: Double inlet left ventricle
DORV: Double outlet right ventricle
ECG: Electrocardiogram
EF: Ejection fraction
eGFR: Estimated glomerular filtration rate
GBPS: Gated blood pool scan
HLHS: Hypoplastic left heart syndrome
ICD: Implantable cardioverter defibrillator
IDR: Incidence density rates
NYHA: New York Heart Association
PAIVS: Pulmonary atresia with intact ventricular septum
SPAS: Systemic-to-pulmonary artery shunt
uAVSD: Unbalanced atrioventricular septal defect
VSD: Ventricular septal defect

## 1 Introduction

“Single ventricle” (SV) heart is a rare anatomical defect accounting for approximately 8% of all patients born with congenital heart disease (CHD) [1]. 10.13039/501100008929SV encompasses a broad range of malformations where septation into a biventricular circulation is not possible because of incomplete development of either a ventricular sinus or an atrioventricular connection, resulting in only one ventricle to support both systemic and pulmonary circulations [2]. Patients with untreated SV lesions have a poor prognosis and frequently suffer from chronic cyanosis, arrhythmia, congestive heart failure, and sudden cardiac death (SCD), usually in childhood [2]. Consequently, where anatomically and physiologically possible, treatment strategies usually involve a series of staged palliative operations, the last of which is known as the Fontan procedure, a palliative surgery that has evolved over time but in all versions connects the systemic venous return to the pulmonary circulation without an intervening pump [2]. The benefits of a Fontan circulation are well-defined, and most patients have improved survival with good functional capacity for the first few decades of life [3]. Yet, as more Fontan recipients enter middle adulthood, a wide array of end-organ complications related to Fontan physiology are observed including cirrhosis, restrictive lung disease, protein-losing enteropathy, chronic venous disease, and other multisystem issues [4]. Pediatric CHD services are sometimes faced with patients who are suboptimal candidates for the Fontan procedure. The most significant contraindications to Fontan palliation include severe ventricular dysfunction, elevated pulmonary vascular resistance (>4 indexed Wood units), and severe hypoplasia of the pulmonary arteries [4,5]. Occasionally, patients in older eras who were “well-balanced” remained unoperated or palliated without a Fontan. These patients frequently had SV subtypes that allowed preferential flow of pulmonary venous return to the systemic circulation, and a degree of pulmonary stenosis that protected the pulmonary arterial system from volume overload [6]. In the current era of aggressive surgical intervention, there is a scarcity of data describing the long-term outcomes of SV patients who have not undergone the Fontan procedure. We aimed to describe the characteristics of adult survivors with SV without Fontan, and to evaluate the long-term outcomes of these patients.

## 2 Methods

### 2.1 Patients and study design

**Fig. 1:**
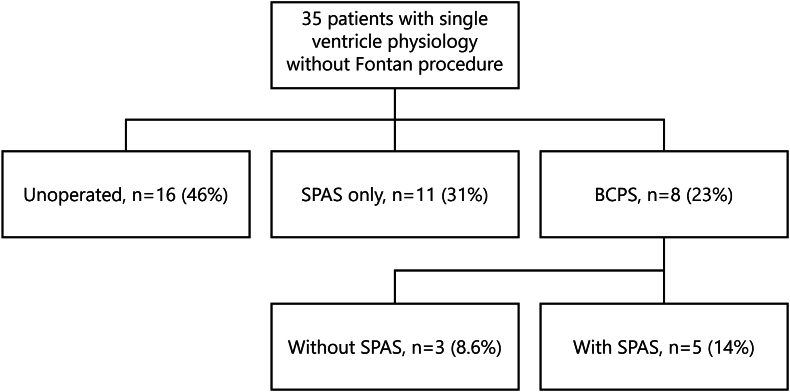
Patient subgroups according to palliation type at latest follow-up. BCPS indicates bidirectional cavopulmonary shunt; SPAS, systemic-to-pulmonary artery shunt.

Two large Adult Congenital Heart Disease (ACHD) centers in Australia (The Royal Melbourne Hospital and Royal Prince Alfred Hospital) participated in this retrospective observational study. Local prospectively maintained ACHD databases were screened for all adult patients (≥16 years of age) with SV diagnoses as classified by the Congenital Heart Surgery Nomenclature and Database Project including hypoplastic left heart syndrome (HLHS), mitral valve atresia, single left ventricle, tricuspid atresia, double inlet left ventricle (DILV), double outlet right ventricle (DORV), pulmonary atresia with intact ventricular septum (PAIVS), and unbalanced atrioventricular septal defect (uAVSD) (Table 1) [7]. Patients who had Fontan physiology at latest follow-up were excluded. The whole cohort was subdivided into three groups according to their underlying physiology at latest follow-up: unoperated group, systemic-to-pulmonary artery shunt (SPAS) only group, and bidirectional cavopulmonary shunt (BCPS) (with or without SPAS) group (Fig. 1). This study was approved by the Human Research Ethics Committees at The Royal Melbourne Hospital and Royal Prince Alfred Hospital (reference no. 57818) and the need for informed consent was waived given the retrospective design of this study.

**Table 1:** Patient characteristics.

	Total (*n* = 35)	Alive (*n* = 26)	Deceased (*n* = 9)	*p*-value
Male, n (%)	16 (46)	11 (42)	5 (56)	0.70
Age at first ACHD visit, years [IQR]	31 [20–40]	22 [19–38]	37 [31–43]	0.10
Cardiac diagnosis at birth, n (%)				
HLHS/mitral atresia	2 (5.7)	2 (7.7)	0 (0.0)	0.41
Double inlet left ventricle	12 (34)	7 (27)	5 (56)	
Double outlet right ventricle	3 (8.6)	3 (12)	0 (0.0)	
Tricuspid atresia	6 (17)	5 (19)	1 (11)	
PAIVS	3 (8.6)	3 (12)	0 (0.0)	
TGA with hypoplastic right ventricle	2 (5.7)	1 (3.8)	1 (11)	
Unbalanced AVSD	6 (17)	5 (19)	1 (11)	
Other complex	1 (2.9)	0 (0.0)	1 (11)	
Left ventricular morphology, n (%)	24 (69)	17 (65)	7 (78)	0.69
Atrial septal defect, n (%)	20 (57)	16 (62)	4 (44)	0.45
Ventricular septal defect, n (%)	28 (80)	20 (77)	8 (89)	0.65
Previous events, n (%)				
Atrial or ventricular arrhythmia	10 (28.6)	4 (15)	5 (56)	0.03
Pacemaker implantation	2 (5.7)	1 (3.8)	1 (11)	0.45
Endocarditis	4 (11)	3 (12)	1 (11)	>0.99
Stroke	3 (8.6)	3 (12)	0 (0.0)	0.55
Pulmonary hypertension	20 (57)	15 (58)	5 (56)	>0.99
NYHA class III or IV, n (%)	4 (12)	4 (15)	0 (0.0)	0.55
Body mass index, kg/m^2^ [IQR]	23 [20–27]	24 [21–28]	19 [17–22]	0.05
O_2_ saturation, % (SD)	83 (6.9)	82 (7.5)	85 (5.3)	0.32
Medications, n (%)				
Beta blocker	7 (21)	3 (12)	4 (50)	0.04
ACE inhibitor or ARB	6 (18)	3 (12)	3 (38)	0.13
Aspirin	12 (35)	11 (42)	1 (13)	0.21
Warfarin	5 (15)	3 (12)	2 (25)	0.57
Diuretic	10 (29)	8 (31)	2 (25)	>0.99
Antiarrhythmic	10 (29)	6 (23)	4 (50)	0.20
Hemoglobin, g/L (SD)	195 (29)	198 (33)	189 (18)	0.46
Platelets, x10^9^/L (SD)	188 (49)	183 (49)	198 (51)	0.50
eGFR <60, n (%)	2 (12)	0 (0.0)	2 (50)	0.04
Electrocardiogram				
Sinus rhythm, n (%)	31 (94)	24 (96)	7 (88)	0.43
Atrial arrhythmia, n (%)	1 (3.0)	1 (4.0)	0 (0.0)	>0.99
QRS >120 ms, n (%)	8 (33)	3 (16)	3 (75)	0.04
Transthoracic echocardiogram				
Moderate/severe ventricular dysfunction (EF <45%), n (%)	2 (6.1)	2 (8.0)	0 (0.0)	>0.99
Valvulopathy (moderate/severe), n (%)				
Mitral regurgitation	8 (24)	6 (24)	2 (25)	>0.99
Tricuspid regurgitation	4 (12)	3 (12)	1 (13)	>0.99
Pulmonary stenosis	12 (36)	9 (36)	3 (38)	>0.99

ACE indicates angiotensin-converting-enzyme; ACHD, adult congenital heart disease; ARB, angiotensin II receptor blockers; AVSD, atrioventricular septal defect; EF, ejection fraction; eGFR, estimated glomerular filtration rate; HLHS, hypoplastic left heart syndrome; PAIVS, pulmonary artery with intact ventricular septum; NYHA, New York Heart Association; TTE, transthoracic echocardiogram.

### 2.2 Data collection

After identification of the study population, clinical charts were reviewed for the study period between May 1981 and December 2020. Baseline data were recorded at the first ACHD clinic visit, and follow-up data were collected from subsequent outpatient visits or hospital admissions. Patient data including cardiac diagnoses, past medical and surgical history, contraindication(s) to Fontan palliation, investigation results at first and latest follow-up, hospital admission data, adverse outcomes, and transplantation status were collected from medical charts. Investigation results including laboratory data, electrocardiogram (ECG), transthoracic echocardiogram, cardiac magnetic resonance imaging (CMR), 6-minute walk test (6MWT), and Holter monitor data were obtained from medical records and verified by study authors (WZ, ML) if deemed unclear or ambiguous. Only moderate or severe valvular stenosis and regurgitation on echocardiogram were considered to be significant valvular disease. The primary endpoint was all-cause mortality. Secondary endpoints included hospitalization for heart failure, new stroke, and new arrhythmia (atrial or ventricular).

### 2.3 Statistical analysis

Continuous variables were assessed for normality using the Kolmogorov-Smirnov test. Data were summarized as frequencies (count, %) for categorical variables, and either mean (± standard deviation) or median (interquartile range) for normal and non-normal continuous variables, respectively. Time-to-event endpoints were depicted using Kapan-Meier curves with 95% confidence intervals (CI), accounting for left-truncation and immortal time bias, with patients censored at the endpoint event or end of follow-up. Fisher's exact test was performed to assess association between baseline characteristics and endpoints. Paired two-sample *t*-test was used to evaluate changes in clinical parameters between first and latest ACHD clinic visits. All tests were two-tailed, evaluated at the 5% significance level. Three continuous variables were prospectively dichotomized based on the following cut-off values: prolonged QRS complex duration (>120 ms), renal impairment (eGFR <60 ml/min/1.73 m^2^), and moderate or severe systemic ventricular dysfunction (ejection fraction [EF] <45%). Participants with missing variables were not included in the count and subsequently were not included in the Fisher's exact test for that variable as a risk factor for endpoint events. Incidence density rates (IDR) were calculated for primary and secondary events. Data analysis was performed using IBM SPSS Statistics 26.0 (IBM Corp. Armonk, New York) with the exception of survival analysis, which was performed using Stata 13.0 (StataCorp. College Station, Texas).

## 3 Results

### 3.1 Patient characteristics

Thirty-five patients with SV physiology without Fontan circulation at latest follow-up were identified. Median age at first ACHD clinic visit was 31 years (IQR: 20–40), with 8 patients over 40 years of age. Table 1, Table 2 summarize baseline patient and surgical characteristics. The most common reasons for not progressing to Fontan palliation were pulmonary vascular disease or pulmonary hypertension (54%, 19/35), patient refusal of Fontan (17%, 6/35), Fontan failure with takedown (14%, 5/35), and hypoplastic pulmonary arteries (11%, 4/35). The most common congenital cardiac diagnoses were DILV (34%, 12/35), followed by tricuspid atresia (17%, 6/35) and uAVSD (17%, 6/35). Left ventricular morphology was most frequently observed (69%, 24/35). Almost half of patients (43%, 15/35) had significant pulmonary stenosis.

**Table 2:** Surgical characteristics.

	Total (*n* = 35)	Alive (*n* = 26)	Deceased (*n* = 9)	*p*-value
Previous palliation, n (%)				
Unoperated	16 (46)	12 (46)	4 (44)	>0.99
Once	4 (11)	3 (11)	1 (11)	>0.99
More than one palliation	15 (43)	11 (42)	4 (44)	>0.99
Age at first palliation, years [IQR]	5.8 [0.01–12]	0.14 [0.01–4.2]	10 [1.0–18]	0.33
Type of palliation, n (%)				
Pulmonary artery banding	2 (5.7)	2 (7.7)	0 (0.0)	>0.99
SPAS only	11 (31)	7 (27)	4 (44)	0.42
BCPS ± SPAS	8 (23)	7 (27)	1 (11)	0.65
BCPS only	3 (8.6)	3 (11.5)	0 (0.0)	0.55
BCPS with SPAS	5 (14)	4 (15)	1 (11)	>0.99
Aortic arch repair	1 (2.9)	1 (3.8)	0 (0.0)	>0.99
Reason for not performing Fontan, n (%)				
Pulmonary vascular disease	19 (54)	16 (62)	3 (33)	
Hypoplastic PA(s)	4 (11)	4 (15)	0 (0.0)	
Atrioventricular valve regurgitation	2 (5.7)	2 (7.7)	0 (0.0)	
Ventricular dysfunction	2 (5.7)	2 (7.7)	0 (0.0)	
High-risk (multiple contraindications)	2 (5.7)	1 (3.8)	1 (11)	
Fontan failure and takedown	5 (14)	4 (15)	1 (11)	
BCPS failure and takedown	1 (2.9)	1 (3.8)	0 (0.0)	
Awaiting heart transplantation	2 (5.7)	1 (3.8)	1 (11)	
Patient refusal	6 (17)	3 (12)	3 (33)	
Unknown	1 (2.9)	1 (3.8)	0 (0.0)	

BCPS indicates bidirectional cavopulmonary shunt; PA, pulmonary artery; SPAS, systemic-to-pulmonary artery shunt.

At first ACHD clinic review, 88% of patients (31/35) were in New York Heart Association (NYHA) Class I or II, with a mean oxygen saturation of 83 ± 6.9% and mean hemoglobin of 195 ± 29 g/L. Previous surgical palliation was performed in 54% of patients (19/35) with 43% (15/35) receiving more than one palliative procedure. Of the subgroups, 46% (16/35) were unoperated, 31% (11/35) were left with SPAS only, and 23% (8/35) were left with BCPS as final palliation. Most unoperated patients had DILV morphology (44%, 7/16), followed by uAVSD (31%, 5/16) and DORV (13%, 2/16). Half of patients with DILV and pulmonary stenosis (50%, 4/8) were unoperated. No differences in baseline characteristics were noted between patients who refused Fontan compared to patients medically deemed to have an absolute contraindication to Fontan.

### 3.2 Survival analysis and mortality predictors

**Fig. 2:**
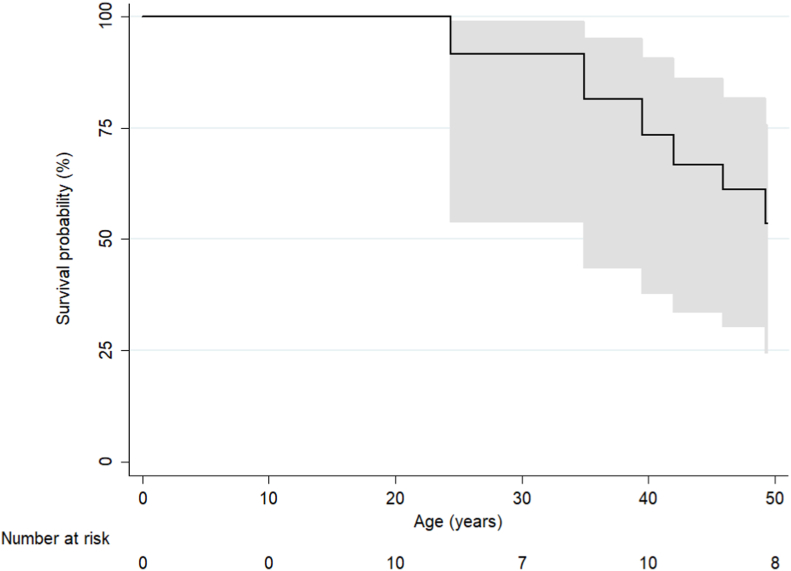
Late survival of whole cohort of SV patients without Fontan from first presentation to ACHD clinic.

Nine (26%, 9/35) patients died during follow-up. The most common causes of death were heart failure (67%, 6/9) and SCD (22%, 2/9) (Table 3). Median age at death was 46 [IQR: 37–52] years. Age-adjusted survival of these adult SV survivors was 73% (95% CI: 38%–91%) and 53% (95% CI: 24%–76%) at 40 and 50 years of age, respectively (Fig. 2). Survival from first ACHD follow-up was 90% (95% CI: 71%–97%) at 5 years and 80% (58–91) at 10 years. Survival between the three palliation subgroups (unoperated, SPAS only, and BCPS ± SPAS) was not significantly different (*p* = 0.28). Compared to those alive, deceased patients were more likely to have baseline eGFR <60 ml/min/1.73 m^2^ (50% [2/4] vs 0% [0/13], *p* = 0.04), baseline QRS >120 ms (75% [3/4] vs 16% [3/19], *p* = 0.04), pre-existing or development of new atrial/ventricular arrhythmia during follow-up (89% [8/9] vs 42% [11/26], *p* = 0.02), baseline lower body mass index (BMI) (19 [IQR: 17–22] vs 24 [21–28] kg/m^2^, *p* = 0.05), and lower systemic ventricular EF on CMR at latest follow-up (30 ± 9.2% vs 51 ± 8.7%, *p* = 0.01).

**Table 3:** Long-term outcomes.

	Total (*n* = 35)	Alive (*n* = 26)	Deceased (*n* = 9)	*p*-value
Age at last follow-up or death, years	42 [28–51]	–	–	–
Follow-up duration, years	10 (8)	–	–	–
Death, n (%) [IDR 1000 person-year]	9 (26) [25]	–	–	–
End-stage heart failure	6 (67)	–	–	–
Sudden cardiac death	2 (22)	–	–	–
Post-operative	1 (11)	–	–	–
Heart-lung transplantation, n (%) [IDR 1000 person-year]	2 (5.7) [5.6]	2 (8.0)	0 (0.0)	>0.99
Any arrhythmia, n (%) [IDR 1000 person-year]	19 (54) [72]	11 (42)	8 (89)	0.02
New atrial or ventricular arrhythmia	15 (43) [55]	10 (39)	5 (56)	0.45
New atrial arrhythmia	14 (40) [46]	10 (39)	4 (44)	>0.99
New ventricular arrhythmia	3 (8.6) [9.6]	2 (7.7)	1 (11.1)	>0.99
Complete heart block	1 (2.9)	1 (3.8)	0 (0.0)	>0.99
DC cardioversion required	10 (29)	6 (23)	4 (44)	0.39
Pacemaker/ICD implantation, n (%) [IDR 1000 person-year]	5 (14.3) [14]	3 (12)	2 (22)	0.59
Pacemaker	3 (8.6)	2 (7.7)	1 (11)	–
ICD	2 (5.7)	1 (3.8)	1 (11)	–
Infective endocarditis, n (%) [IDR 1000 person-year]	3 (8.6) [8.4]	3 (12)	0 (0.0)	0.55
Stroke, n (%) [IDR 1000 person-year]	6 (17) [17]	4 (15)	2 (22)	0.64
Any hospital admission, n (%) [IDR 1000 person-year]	23 (66) [64]	15 (58)	8 (89)	0.14
Hospital admission for heart failure, n (%) [IDR 1000 person-year]	12 (34) [34]	6 (23)	6 (67)	0.03

DC indicates direct current; ICD, implantable cardioverter defibrillator; IDR, incidence density rate.

### 3.3 Heart failure and heart-lung transplantation

**Fig. 3:**
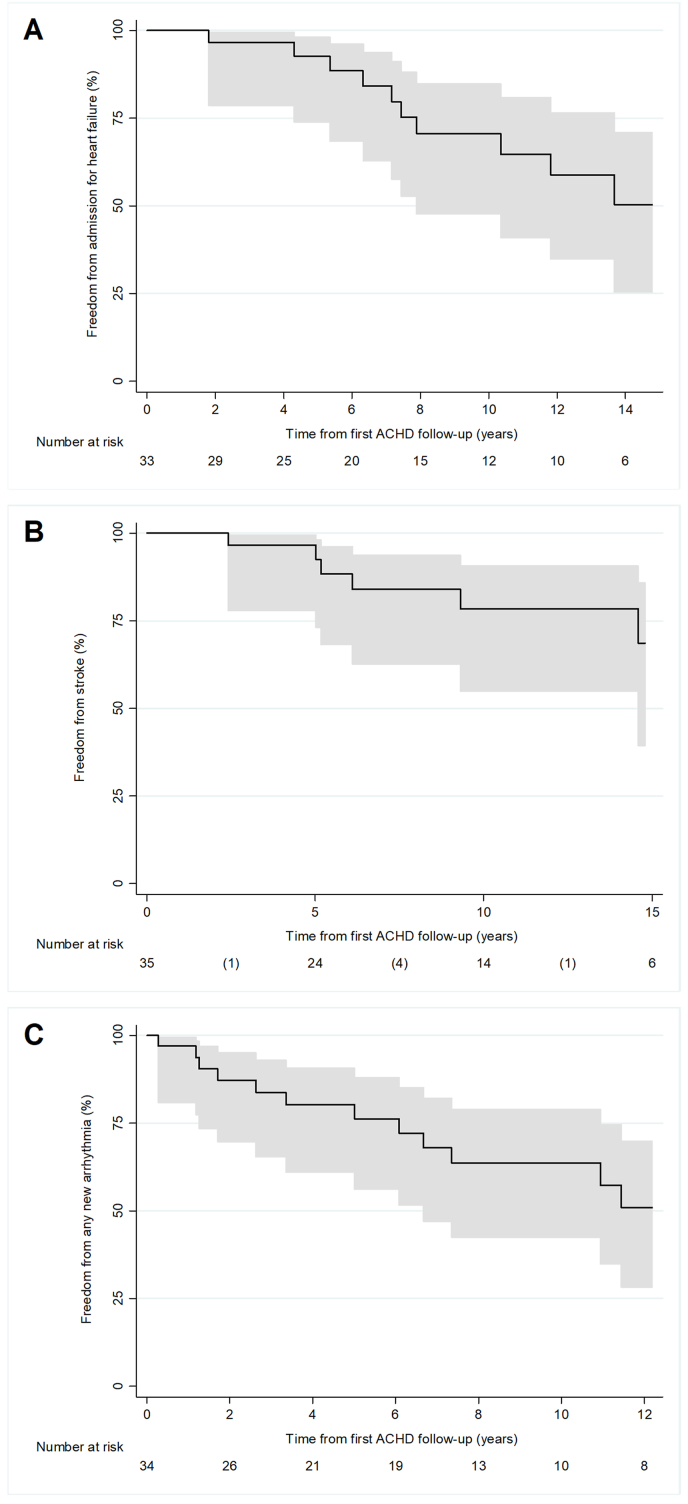
Freedom from cardiovascular events for whole cohort of SV patients without Fontan from first ACHD follow-up: (A) admission for heart failure, (B) new stroke, and (C) any new arrhythmia.

Heart failure-related outcomes were frequently observed. One-third of all patients (34%, 12/35) were admitted to hospital for heart failure and 20% of patients (7/35) had more than one such admission. Patients requiring admission for heart failure were older on first ACHD visit (37 ± 11 vs 28 ± 12 years, *p* = 0.04) and more likely to have baseline QRS >120 ms (57% [4/7] vs 13% [2/16], *p* = 0.05). Compared to first ACHD visit, more patients were managed on beta blockers (46% [16/35] vs 21% [7/34], *p* = 0.04) and diuretics (63% [22/35] vs 29% [10/34], *p* < 0.01) at latest follow-up. There was no increase in the proportion of patients with moderate or severe ventricular dysfunction on echocardiography between first and latest follow-up (6.1% [2/33] vs 12% [4/33], *p* = 0.67) (Table 4). Freedom from heart failure hospitalization was 93% (95% CI: 74%–98%) and 65% (41–81) at 5 and 10 years from first ACHD follow-up, respectively (Fig. 3). Patients requiring hospitalization for heart failure had higher ventricular end diastolic volume index (272 [IQR: 185–386] vs 110 [76–221] ml/m^2^, *p* = 0.05). Four patients (11%, 4/35) were referred for heart transplantation. Two patients (5.7%, 2/35) received combined heart-lung transplantation for severe irreversible pulmonary vascular disease with decline in functional status to NYHA Class III or IV. After heart-lung transplantation, both patients improved to NYHA Class I or II at latest follow-up. One patient died while on the transplant waiting list and one patient was still awaiting transplantation at time of data analysis.

**Table 4:** Comparison of clinical, laboratory, and imaging parameters between first and latest follow-up.

	First follow-up (*n* = 35)	Latest follow-up (*n* = 35)	*p*-value
NYHA class III or IV, n (%)	4 (12)	9 (27)	0.22
Body mass index, kg/m^2^ [IQR]	23 [19–27]	22 [20–27]	0.67
O_2_ saturation, % (SD)	83 (6.9)	80 (8.8)	0.10
Medications, n (%)			
Beta blocker	7 (21)	16 (46)	0.04
ACE inhibitor/ARB	7 (20)	9 (25.7)	0.78
Aspirin	12 (35)	15 (43)	0.62
Warfarin	5 (15)	10 (29)	0.24
DOAC	0 (0.0)	2 (5.7)	0.49
Diuretic	10 (29)	22 (63)	0.01
Antiarrhythmic	10 (29)	14 (40)	0.45
Hemoglobin, g/L (SD)	195 (29)	183 (31)	0.16
Platelets, x10^9^/L (SD)	188 (49)	185 (68)	0.86
eGFR <60, n (%)	2 (12)	6 (23)	0.45
Electrocardiogram	*n* = 33	*n* = 24	
Sinus rhythm, n (%)	31 (94)	19 (79)	0.19
Atrial arrhythmia, n (%)	1 (3.0)	3 (13)	
Complete heart block, n (%)	0 (0.0)	1 (4.2)	
Paced rhythm, n (%)	1 (3.0)	1 (4.2)	
QRS duration, ms (SD)	113 (17)	135 (34)	<0.01
Transthoracic echocardiogram	*n* = 33	*n* = 33	
Moderate/severe ventricular dysfunction (EF <45%), n (%)	2 (6.1)	4 (12)	0.67
Valvulopathy (moderate/severe), n (%)			
Mitral regurgitation	8 (24)	10 (30)	0.78
Tricuspid regurgitation	4 (12)	6 (18)	0.73
Pulmonary regurgitation	0 (0.0)	2 (6.1)	0.49
Pulmonary stenosis	12 (36)	13 (39)	>0.99
Six-minute walk test	*n* = 10	*n* = 19	
Pre-exercise heart rate, (SD)	87 (13)	81 (13)	0.17
Maximum heart rate during exercise, (SD)	119 (21)	107 (19)	0.10
Baseline O_2_ saturation, % (SD)	84 (6.3)	82 (4.9)	0.40
O_2_ saturation at 1-min recovery, % (SD)	78 (9.0)	79 (6.1)	0.63
Lowest O_2_ saturation during exercise, % (SD)	67 (11)	65 (6.5)	0.63
Distance, meters [IQR]	–	390 [368–434]^a^	–
Holter monitor	–	*n* = 16	
Total ventricular ectopic beats [IQR]	–	1241 [368–5884]	–
Non‒sustained ventricular tachycardia, n (%)	–	7 (44)	–
Sustained ventricular tachycardia, n (%)	–	0 (0.0)	–
Cardiac MRI	–	*n* = 17	
SV ejection fraction, % [IQR]	–	47 [42–58]	–
SV end diastolic volume index, ml/m^2^ [IQR]	–	190 [89–272]	–
SV end systolic volume index, ml/m^2^ [IQR]	–	94 [39–139]	–

ACE indicates angiotensin-converting enzyme; ACHD, Adult Congenital Heart Disease; ARB, angiotensin II receptor blockers; DOAC, direct-acting oral anticoagulant; ECG, electrocardiogram; GFR, glomerular filtration rate; SV, single ventricular; NHYA, New York Heart Association.

a Only 10 patients had a 6MWT distance recorded.

### 3.4 Stroke

From birth to latest ACHD follow-up, seven patients had a stroke (20%, 7/35). Three patients had a stroke prior to first ACHD visit (8.6%, 3/35), and six new strokes were observed after first ACHD visit (17%, 6/35), of which two occurred in those with a previous stroke. Freedom from new stroke from first ACHD visit was 93% (95% CI: 73%–98%) and 78% (55–91) at 5 and 10 years of age, respectively (Fig. 3). The prognosis of patients with stroke was poor with mortality of 33% (2/6). One patient suffered a hemorrhagic stroke while on warfarin, and the other five patients were not on anticoagulant treatment prior to their ischemic stroke. In two patients (33%, 2/6), the stroke was related to a new episode of supraventricular arrhythmia. Patients with stroke were more likely to have atrial isomerism (50% [3/6] vs 6.9% [2/29], *p* = 0.03), and a BCPS with SPAS (50% [3/6)] vs 6.9% [2/29], *p* = 0.03).

### 3.5 Arrhythmias and devices

Seven patients had a diagnosis of arrhythmia (20%, 7/35) prior to first ACHD visit, including atrial arrhythmia in six patients and complete heart block in one patient. A new episode of atrial fibrillation or flutter was seen in 40% of patients (14/35), with eight patients undergoing electrophysiology studies (Fig. 3). Atrial arrhythmias were frequently observed in patients who developed other complications. Among those who developed new atrial arrhythmia, 21% had a stroke (3/14), 43% had been hospitalized for heart failure (6/14), and 36% died (5/14). Two patients with new atrial arrhythmia were commenced on a direct-acting oral anticoagulant due to patient preference and neither have had a stroke during follow-up. New sustained ventricular arrhythmias were detected in three patients (8.6%, 3/35). A total of four epicardial pacemakers were implanted. Two implantable cardioverter defibrillators (ICD) were offered, one for primary prevention and one for secondary prevention. Patients who had pre-existing or new atrial/ventricular arrhythmia were more likely male (63% [12/19] vs 25% [4/16], *p* = 0.04) and had atrial isomerism (26% [5/19] vs 0% [0/16], *p* = 0.05). Freedom from any new arrhythmia was 76% (95% CI: 56%–88%) at 5 years and 57% (35–75) at 10 years from first ACHD follow-up (Fig. 3). At latest follow-up, patients with any arrhythmia had higher CMR-derived ventricular end diastolic volume (272 [IQR: 201–386] vs 110 [76–196] ml/m^2^, *p* = 0.02) and ventricular end systolic volume (139 [100–199] vs 43 [28–71] ml/m^2^, *p* = 0.02) indices and a trend towards lower ventricular EF (44% [33–48] vs 54% [46–64], *p* = 0.06) compared to those without any arrhythmia. Those with any arrhythmia were also more likely to have significant mitral regurgitation (47% [9/19] vs 7.1% [1/14], *p* = 0.02) on echocardiogram.

### 3.6 Infectious complications

Nine patients (26%, 9/35) developed major infections requiring hospital admission. Endocarditis was diagnosed in three (8.6%, 3/35) patients, and two cases of brain abscess were observed. There was no difference in baseline oxygen saturation level between those who developed a brain abscess and those who did not.

### 3.7 Surgical or transcatheter interventions

Twenty-five interventions were performed across 16 patients (46%, 16/35) with the most frequent being transcatheter procedures (*n* = 15), pacemaker or ICD implantation (*n* = 5), and cardiac surgery (*n* = 5) (including BCPS and pulmonary artery banding in one patient). Transcatheter procedures included pulmonary artery or shunt angioplasty (*n* = 9), pulmonary valvuloplasty (*n* = 2), major aortopulmonary collateral artery embolization (*n* = 2), bronchial artery embolization (*n* = 1), and device closure of veno-venous collateral (*n* = 1).

### 3.8 Status at latest follow-up

Of the 35 patients included, 26 (74%) were alive at time of analysis with a median age of 37 years [IQR: 27–51, maximum 68] and the majority in NYHA Class I or II (76%, 19/25). Mean follow-up time from first ACHD clinic review was 10 ± 8.3 years. Table 4 summarizes changes in clinical, laboratory, imaging, and other investigation parameters during follow-up. Compared to first follow-up, a significant decrease was observed in albumin levels (37 ± 4.8 vs 41 ± 5.7 g/L, *p* = 0.01) at latest follow-up, whereas serum urea (8.3 ± 4.9 vs 5.5 ± 1.5 mmol/L, *p* = 0.01) and QRS duration (135 ± 34 vs 113 ± 17 ms, *p* < 0.01) increased. No patients had a diagnosis of protein-losing enteropathy.

ECG at latest clinic visit showed sinus rhythm in 79% of patients (19/24), atrial arrhythmia in 13% (3/24), complete heart block in 4% (1/24), and paced rhythm in 4% (1/24). A total of 16 patients (46%, 16/35) received Holter monitoring at a median time of 4.4 years (IQR: 2.3–9.6) from first clinic visit. Of these patients, a high ventricular ectopic burden (>10 ectopies per hour) was noted in 56% (9/16), non-sustained ventricular tachycardia in 44% (7/16), and sinus pauses in 25% (4/16). No patients had Holter-confirmed sustained ventricular tachycardia (>30 seconds).

Over half of patients (54%, 19/35) underwent a 6MWT at a median time of 9.2 years (IQR: 5.3–12) from first clinic visit. Mean O_2_ saturation was 82 ± 4.9% at baseline, a nadir of 65 ± 6.5% during exercise, and 79 ± 6.1% at 1-min recovery. A total of 10 patients (53%, 10/19) had a 6MWT distance recorded with a median of 390 meters (IQR: 368–434). Of these patients, 6 were in NYHA Class II and 4 were in NYHA Class III at the time of testing, and there was no significant difference in 6MWT distances between the two functional class groups, respectively (390 [IQR: 373–469] vs 383 [304–442] meters, *p* = 0.610).

Transthoracic echocardiogram results were available for 94% of patients (33/35) of which 75% (25/33) were performed at latest clinic visit and 25% (8/33) within two years of latest clinic visit. Four patients (12%, 4/33) had moderate or severe ventricular dysfunction. Significant valvular disease was observed in 66% of patients (22/33): mitral regurgitation in 30% (10/33), tricuspid regurgitation in 18% (6/33), pulmonary stenosis in 39% (13/33), and pulmonary regurgitation in 6.1% (2/33). CMR was performed for half of the study cohort (49%, 17/35) at a median time of 4.6 years (IQR: 2.0–8.0) from latest clinic visit. The median ventricular EF was 47% (IQR: 42–58).

## 4 Discussion

We report one of the few contemporary long-term follow-up series of adult survivors with SV who have not received Fontan palliation. Our study demonstrates that patients may reach adulthood, albeit with a high mortality and significant burden of heart failure and arrhythmias. Nearly half of patients will develop a new atrial or ventricular arrhythmia by 10 years from first ACHD clinic visit. Pre-existing or development of new atrial/ventricular arrhythmia, QRS complex prolongation, renal impairment, and lower BMI were associated with higher mortality.

SV comprises a heterogenous group of cardiac morphologies and in the contemporary era, most patients will have had some form of surgical palliation [8]. Consequently, the natural prognosis of unoperated patients with SV is difficult to accurately ascertain [9]. Historical studies have reported exceedingly high mortality rates [[10], [11], [12], [13]]. Late survival without any operative intervention is only possible in a small number of patients with balanced systemic and pulmonary circulations [6,14,15]. DILV anatomy with some degree of pulmonary stenosis has been described to confer survival benefit [6]. In our cohort of long-term survivors, we observed a high proportion of patients with such characteristics and the majority were unoperated. Notably, several unoperated patients in our cohort survived into the fifth, up to even the seventh, decade of life with good functional status, corroborating with a collection of case reports that those with an adequately balanced physiology can live through middle adulthood with a reasonable quality of life [6,14,15].

Patients left with non-Fontan palliation are known to have substantial long-term mortality [10,[16], [17], [18], [19], [20], [21]]. We report overall survival of 90% at 5 years and 80% at 10 years of follow-up. In a Spanish multicenter study, Buendía-Fuentes and colleagues described similar survival of 86% at 5 years and 74% at 10 years from first ACHD follow-up for a cohort of 146 patients with SV without Fontan palliation [22]. Of recent reports, patient characteristics of this Spanish cohort were most comparable to those of our group. Between the two studies, patients were predominantly excluded from Fontan completion due to high-risk contraindications (e.g. hypoplastic pulmonary arteries, high pulmonary vascular resistance, and ventricular dysfunction) as opposed to historical reports where patients would have been, for the large part, suitable Fontan candidates with contemporary care [22]. Interestingly, the mean age of both cohorts at first ACHD clinic visit were considerably older (33 ± 11 years for the Spanish cohort and 31 ± 12 years for our cohort) suggesting there is a subgroup of patients with delayed transition to tertiary ACHD care, either because they were managed outside of ACHD centers due to their poor perceived prognosis, or lost to follow-up and referred later in life [9,22]. For this cohort of adult single ventricle survivors without Fontan palliation, it appears that functional capacity is limited, cardiovascular complications are common, and barriers exist to accessing specialist ACHD care [16,17,[22], [23], [24], [25]].

Prognostication is potentially valuable for this high-risk cohort. We identified four variables associated with long-term mortality: eGFR <60 ml/min/1.73 m^2^, lower BMI, pre-existing or development of new atrial or ventricular arrhythmia, and QRS complex >120 ms. These readily available variables have been previously described as mortality predictors in other complex CHDs, however data pertaining to adverse prognostic factors specific to SV patients who do not undergo Fontan palliation are lacking [[26], [27], [28], [29], [30], [31]]. Renal impairment is commonly related to acquired heart failure and frequently observed in cyanotic patients and two-thirds of those with Eisenmenger syndrome [28]. Moderate or severe renal dysfunction is associated with up to a three-fold increase in mortality for ACHD patients, irrespective of ventricular dysfunction, and has been specifically identified as an independent mortality predictor in SV patients without Fontan [22,28]. Renal impairment is also recognized to increase perioperative risk and it is possible that these patients were less likely to be offered further palliative surgery or heart transplantation [[31], [32], [33]]. Routine, periodic screening of renal function in this cohort should be performed to identify those who may benefit from more comprehensive assessment. Similarly, lower BMI and weight loss have been reported to predict mortality especially in patients with complex and symptomatic ACHD [27]. To our knowledge, this is the first study to observe this association specifically in SV patients without Fontan palliation. This may relate to reduced metabolic reserve, wasting, and cardiac cachexia in the context of a consuming chronic state [27,34,35]. Whether SV patients would benefit from thorough nutrition evaluation and optimization of weight and BMI is unknown.

Arrhythmia is associated with late morbidity in SV patients without Fontan and several small observational experiences have reported that up to one-third may develop new sustained atrial flutter or fibrillation over 10 years of follow-up [[22], [23], [25]]. We noted a high burden of new atrial and ventricular arrhythmias in our cohort which often coincided with features of increased volume loading and ventricular dysfunction, and ultimately, was associated with substantial mortality. Corroborating other reports, we also identified QRS prolongation to be a risk factor for new atrial arrhythmia and mortality in patients with SV [[22], [29], [30]]. Retrospective studies, including ours, have described several cases where a new episode of atrial arrhythmia was directly implicated in cardioembolic stroke [22]. Although this association did not reach significance in our analysis, these patients with new stroke have a poor prognosis with mortality over 30% [22]. Some studies have reported SCD as the leading cause of mortality in these SV patients, yet rates of detected sustained ventricular arrhythmias were variable from 2% to 20% [[6], [22], [23]]. Patient selection for primary prevention of SCD in this cohort is challenging with no evidence-based guidelines available. Close monitoring for arrhythmias in SV patients without Fontan, particularly in those with ventricular dilatation and/or ventricular dysfunction, is crucial. Treating physicians should have a low threshold when considering initialization of anti-thrombotic therapy in those with atrial arrhythmia.

It is well-established that heart failure is prevalent in SV patients without Fontan palliation and carries a high mortality risk, however few studies have evaluated the burden of heart failure hospitalizations, which can greatly contribute to reduced quality of life [22]. Contrary to existing studies where SCD was the most common cause of mortality, we demonstrated decompensated heart failure to be the most common cause in our series, accounting for two-thirds of deceased patients [6,22,23]. We observed heart failure hospitalization in one-third of our group, at incidence rates consistent with those reported by Buendía-Fuentes et al. [22]. For those left with a SPAS only, there are indications this subgroup may have worse ventricular function, possibly related to greater volume loading of their systemic ventricle [10,18,22,23]. We noted a trend towards increased heart failure hospitalization in those with SPAS as final palliation, although this did not reach significance (*p* = 0.06). Heart transplantation is the last-line strategy for interstage attrition for patients with SV deemed unsuitable for Fontan [36]. The low rates of heart transplantation across several studies, including ours, underscore the ongoing issue of donor shortages observed not only in Australia but in other international contexts too [22,23,37].

### 4.1 Study limitations

Survivorship bias must be considered for our study as we only report adult patients greater than 16 years of age. The true natural fate of this population may be less favorable than described in retrospective observational studies [6,9,11,22,23]. Ideally, historical comparisons would be undertaken between individuals, with the same contraindications, who underwent Fontan palliation (current surgical era cohort) and those who did not (our cohort) to understand whether Fontan palliation improves complications and mortality. This should be considered in future studies. Only a small number of patients (n = 9) had a 6-minute walk test distance at baseline so we were unable to analyze functional capacity over time in our cohort. Finally, due to the retrospective nature of this study, clinical characteristics and outcomes data were extracted as documented in medical charts, and minor inconsistencies in how these variables were defined should be acknowledged.

## 5 Conclusion

Long-term survival in patients with SV without Fontan palliation is possible, albeit with a substantial burden of cardiovascular complications, especially arrhythmia and heart failure hospitalization, leading to late morbidity. Pre-existing or new arrhythmia, QRS complex prolongation, renal impairment, and lower BMI were associated with higher mortality.

## Declaration of competing interest

The authors declare the following financial interests/personal relationships which may be considered as potential competing interests: Melissa Lee holds a Medical Research Future Fund (MRFF) Investigator Grant (1197307). Yves d’Udekem is a Deputy Editor, and David Celermajer and Igor Konstantinov are Associate Editors for IJC-CHD.
